# Coherent imaging at the diffraction limit

**DOI:** 10.1107/S1600577514015343

**Published:** 2014-08-27

**Authors:** Pierre Thibault, Manuel Guizar-Sicairos, Andreas Menzel

**Affiliations:** a Department of Physics and Astronomy, University College London, UK; b Paul Scherrer Institut, 5232 Villigen PSI, Switzerland

**Keywords:** diffraction-limited synchrotron sources, coherent diffraction, imaging, ptychography

## Abstract

X-ray ptychography, a scanning coherent diffractive imaging technique, holds promise for imaging with dose-limited resolution and sensitivity. If the foreseen increase of coherent flux by orders of magnitude can be matched by additional technological and analytical advances, ptychography may approach imaging speeds familiar from full-field methods while retaining its inherently quantitative nature and metrological versatility. Beyond promises of high throughput, spectroscopic applications in three dimensions become feasible, as do measurements of sample dynamics through time-resolved imaging or careful characterization of decoherence effects.

## Abstract 2

The foreseen increase in coherent flux provided by diffraction-limited storage rings will allow ptychography to reach unprecedented throughput while retaining its inherently quantitative nature and metrological versatility.

## 1. Introduction   

From their discovery to this day, X-rays have been used for imaging. Defined broadly as techniques that permit characterization of the spatial distribution of matter, imaging encompasses fields from medical applications to X-ray microscopy and even crystallography. X-rays are valued for their penetration power, their specificity of contrast, and their short wavelength underpinning the potential for high-resolution microscopy.

X-ray microscopy exists in many flavors. Most common are full-field configurations, in which the image is either projected onto the detector or magnified by means of an objective lens, and scanning-probe microscopes, in which focusing optics create a tiny probe through which the sample is scanned. Both synchrotron- and laboratory-based X-ray microscopes can routinely reach a few tens of nanometers in resolution. Most are fitted with rotation stages for tomography and specialized sample environments that allow specimens to remain close to their native condition, to be characterized *in operando*, or to be manipulated *in situ*.

Originally motivated by limitations of image-forming optics, alternative microscopy approaches have been developed that harness the coherent flux that became available at increasingly brilliant X-ray sources. In one form or another, all techniques that depend on coherence aim to encode sample information onto a well defined carrier, thereby making it possible to model and decode the output. If based on interferometric or holographic approaches or on the encoding of sample information in the Fraunhofer far-field, which comprises the set of methods that is frequently called coherent diffractive imaging (CDI), these techniques exploit coherence in order to determine the samples’ complex-valued index of refraction, *n* = 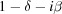. Thus, coherence allows both absorption and phase to be measured quantitatively and in parallel.

This paper is mainly focused on the status and future of ptychography, a member of the CDI family that has attracted particular interest in the last years. Its working principles and the current state of the art are described in the following section. We offer our thoughts on the future of the technique in view of projected technological improvements in §3. In particular, we assess the impact, the potential and the challenges of diffraction-limited storage rings, which promise coherent fluxes that are orders of magnitude higher than available today. Could diffraction-limited sources lead to diffraction-limited imaging?

## 2. Ptychography   

The underlying principle of any CDI technique is to tap into the special properties of coherent fields to bypass or relax experimental constraints. Most CDI techniques are qualified as ‘lensless’ because they do away with image-forming optics, which typically have at least one limitation among low efficiency, strong aberrations and low numerical aperture. In effect, the lens is replaced by a mathematical algorithm, whose task is the reconstruction of the sample’s transmission function from the diffraction measurement. Since only the wavefield’s squared amplitude, *i.e.* the intensity, is measurable, the phase part cannot be determined directly and, as in crystallography, the image reconstruction process is essentially a phase retrieval problem. Fortunately the phase recovery process can be made tractable by ensuring, for instance, that the sample is isolated and its far-field pattern sampled finely enough, which is the ‘canonical’ case of CDI, called diffraction microscopy or single-shot diffractive imaging (Miao *et al.*, 1999 ▶; Chapman & Nugent, 2010 ▶), or that there is a well defined scatterer acting as a reference nearby as in Fourier transform holography (Eisebitt *et al.*, 2004 ▶; Marchesini *et al.*, 2008 ▶). A third possibility is illuminating the sample with a structured, most frequently confined illumination and measuring the resulting diffraction patterns for multiple positions of the sample with respect to this illumination while ensuring some degree of overlap of the illumination between different acquisitions. This approach is called ptychography.

Ptychography was first introduced in the early 1970s to increase the resolution of electron microscopes (Hegerl & Hoppe, 1970 ▶). Despite recent successes with electron beams (Nellist *et al.*, 1995 ▶; Putkunz *et al.*, 2012 ▶; Humphry *et al.*, 2012 ▶), it is nowadays most actively used and developed for X-ray and visible radiation. With its probe swept over the sample, its mode of operation is similar to scanning transmission microscopy, with the notable exception that a full diffraction pattern is measured at each point. Through phase retrieval the resolution of the sample image can be much finer than the dimensions of the probe and the scanning step size. Furthermore, the collected dataset is typically sufficiently redundant to permit recovery of both the sample’s complex-valued transmission function and the profile of the incoming wavefield, *i.e.* there is no need to isolate the sample or to have a ‘clean’ and well characterized incident beam.

**Figure 1:**
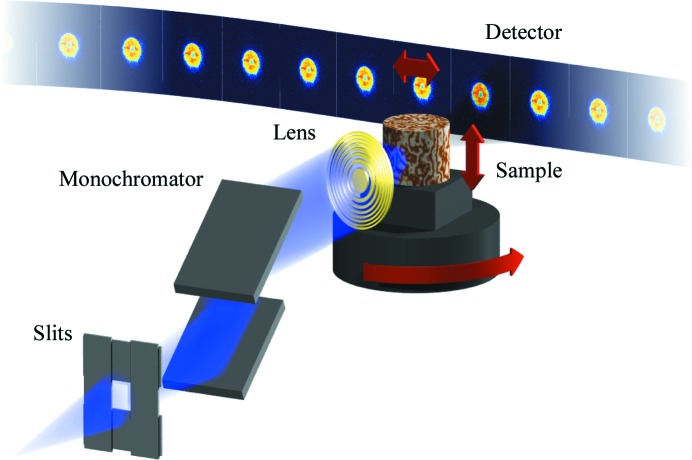
Schematic of a typical ptychography set-up. The X-ray beam generated by the insertion device is filtered spectrally with a monochromator, admitted through one or multiple beam-defining apertures, which act as a coherence filter as well, and focused on the sample. For each position of the sample in the beam, *i.e.* lateral displacement and rotation, the intensity of the scattered X-ray wave is measured in the far-field using a pixel array detector.

A typical X-ray set-up used to measure a ptychographic dataset is shown in Fig. 1 ▶. Assuming for now that the incident beam is constant in time and fully coherent and that the detector collects artifact-free patterns, the most important parameters to consider for a well conditioned reconstruction of a dataset are the scanning pattern and the sampling of the speckle pattern. The positions of the probe on the sample must be chosen in such a way that the probe footprint overlaps. If the conditions for reconstructibility are satisfied, the image quality, *i.e.* resolution and contrast, is expected to depend primarily on the total fluence through the sample, which is spatially varying itself. In practice, many parameters enter into play, and it was recently shown that tailoring the illumination could lead to improvements in reconstruction signal-to-noise ratio (Guizar-Sicairos *et al.*, 2012 ▶; Maiden *et al.*, 2013 ▶). Yet, what precisely are optimal conditions to obtain the best reconstruction in a given experimental setting remains subject to further research.

**Figure 2:**
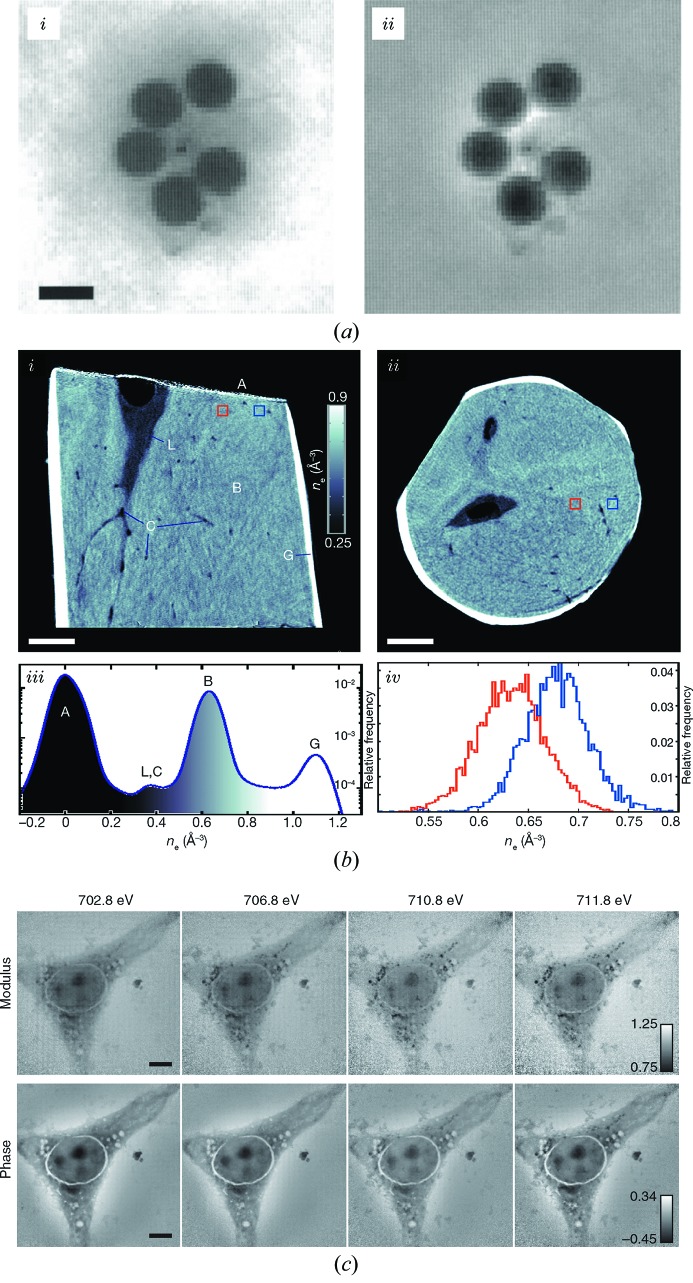
Milestones in the progression of X-ray ptychography. (*a*) An X-ray demonstration of Wigner distribution deconvolution; shown are the amplitude (i) and phase (ii) of the transmission function of a set of latex spheres imaged with 3.1 nm radiation. [Reproduced from Chapman (1997 ▶).] The scale bar represents 0.5 µm. This early work already included the simultaneous reconstruction of the illumination function, including aberration effects and partial coherence effects (Chapman, 1996 ▶). (*b*) An early demonstration of X-ray ptychography, which was used as input to a tomographic reconstruction of a cylinder of murine femur. [Reproduced from Dierolf *et al.* (2010*a* ▶).]. (i) Cuts parallel and (ii) perpendicular to the axis of rotation. In both cases, the scale bar marks 5 µm. Such tomographic reconstructions are particularly reliable for quantitative density estimation. (iii) A histogram of the electron density of the entire tomogram and (iv) of two subvolumes of 1 µm^3^ each, demonstrating on such reduced spatial resolution a density specificity of ∼10^−3^ Å^−3^. (*c*) Demonstration of spectro-ptychography. Shown are both amplitude and phase images of a Balb/3T3 mouse fibroblast doped with cobalt ferrite (CoFe_2_O_4_) nanoparticles, whose contrast clearly vary upon scanning through the Fe *L*_III_-edge. [Reprinted by permission from Macmillan Publishers Ltd: *Nature Communications* (Maiden *et al.*, 2013 ▶), copyright 2013.]

Reconstruction techniques fall into three main categories. The first one, which requires a very tight scanning grid, allows a one-step reconstruction called Wigner distribution deconvolution (Bates & Rodenburg, 1989 ▶; Rodenburg & Bates, 1992 ▶; McCallum & Rodenburg, 1993 ▶; Chapman, 1996 ▶, 1997 ▶), Fig. 2(*a* ▶). More popular nowadays, because of significantly less stringent requirements on the measurements, are iterative projection-based algorithms (Faulkner & Rodenburg, 2004 ▶; Thibault *et al.*, 2009 ▶; Maiden & Rodenburg, 2009 ▶) similar in essence to those used for single-shot imaging (Fienup, 1978 ▶; Elser, 2003 ▶; Luke, 2005 ▶). Finally, non-linear optimization algorithms (Guizar-Sicairos & Fienup, 2008 ▶), and in particular maximum-likelihood optimization, which include noise statistics, have been shown to improve reconstruction, typically as final refinement (Thibault & Guizar-Sicairos, 2012 ▶; Godard *et al.*, 2012 ▶).

The result of a ptychographic reconstruction is, neglecting recent new flavors discussed below, two complex-valued arrays, one representing the amplitude and phase of the incident wavefield, the other representing the transmission function of the sample as a map of the index of refraction projected along the propagation direction. There are a few ways to assess the quality of a reconstruction, the most unambiguous being the Fourier shell correlation method, already commonly used for transmission electron microscopy (van Heel & Schatz, 2005 ▶).

### 2.1. Applications   

Once shown to work reliably in two dimensions (Rodenburg *et al.*, 2007 ▶; Thibault *et al.*, 2008 ▶; Giewekemeyer *et al.*, 2010 ▶; Dierolf *et al.*, 2010*b* ▶; Schropp *et al.*, 2011 ▶), ptychography quickly embraced the third dimension (Dierolf *et al.*, 2010*a* ▶; Guizar-Sicairos *et al.*, 2011 ▶; Peterson *et al.*, 2012 ▶), Fig. 2 ▶(*b*) ▶, proving a valuable tool to produce quantitative high-resolution maps of a sample’s electron density (Diaz *et al.*, 2012 ▶).

**Figure 3:**
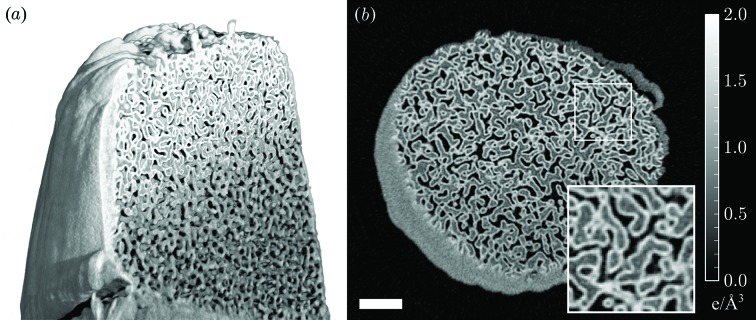
X-ray ptychographic tomography of a nanoporous glass sample. (*a*) Rendering of three-dimensional reconstruction with 22 nm resolution shows a gradient of the thickness of the Ta_2_O_5_ conformal coating in the axial direction. (*b*) Axial section with a clear differentiation between air, glass and the conformal coating. The scale bar is 1 µm. The inset in (*b*) corresponds to the 1.5 µm × 1.5 µm region indicated with a white rectangle.

An example from a recent measurement is shown in Fig. 3 ▶. The sample is based on nanoporous glass, coated with ∼37 nm of Ta_2_O_5_, which provides a radiation-tolerant three-dimensional test sample with good electron density contrast. The sample preparation and measurement and reconstruction protocol are detailed by Holler *et al.* (2014 ▶) with the difference that the scanned field of view was larger, namely 10 µm × 10 µm, and an Eiger detector (Dinapoli *et al.*, 2010 ▶) was used with 0.1 s exposure time, which allowed the acquisition of 720 projections in 8.5 h. The isotropic three-dimensional resolution was determined by Fourier shell correlation with a half-bit criterion (Vila-Comamala *et al.*, 2011 ▶) to be 22 nm. This corresponds to an imaging rate around 3000 resolution elements per second. An imaging rate one order of magnitude higher was demonstrated recently for a large two-dimensional reconstruction (Guizar-Sicairos *et al.*, 2014 ▶).

A theoretical bound in the achievable contrast and spatial resolution can be computed from the knowledge of the number of incident photons involved in the experiment. This fluence is readily computed from the high-resolution intensity profile of the incident illumination recovered from the data.

The tomographic dataset, which was analyzed to yield Fig. 3 ▶, is composed of 720 individual two-dimensional projections, each obtained with a total fluence of about 8.5 × 10^6^ photons µm^−2^. For a reconstruction pixel size of 10 nm × 10 nm, one finds from this number that the effective dynamic range in contrast is ∼30σ, *i.e.* at most 30 ‘discernible levels’. A three-dimensional equivalent of fluence can be computed for the tomographic reconstruction, giving between 200 and 300 photons per 10 nm voxel. The total amount of information on the sample carried by these photons has not yet been quantified rigorously, but simulations indicate that dose fractionation principles (McEwen *et al.*, 1995 ▶) do apply to ptychography. Consequently, the need to acquire multiple diffraction patterns does not necessarily increase the dose imparted on the specimen. Rather, many low-signal diffraction patterns can reliably be reconstructed as long as the physical and noise models are faithfully integrated in the reconstruction through likelihood optimization.

**Figure 4:**
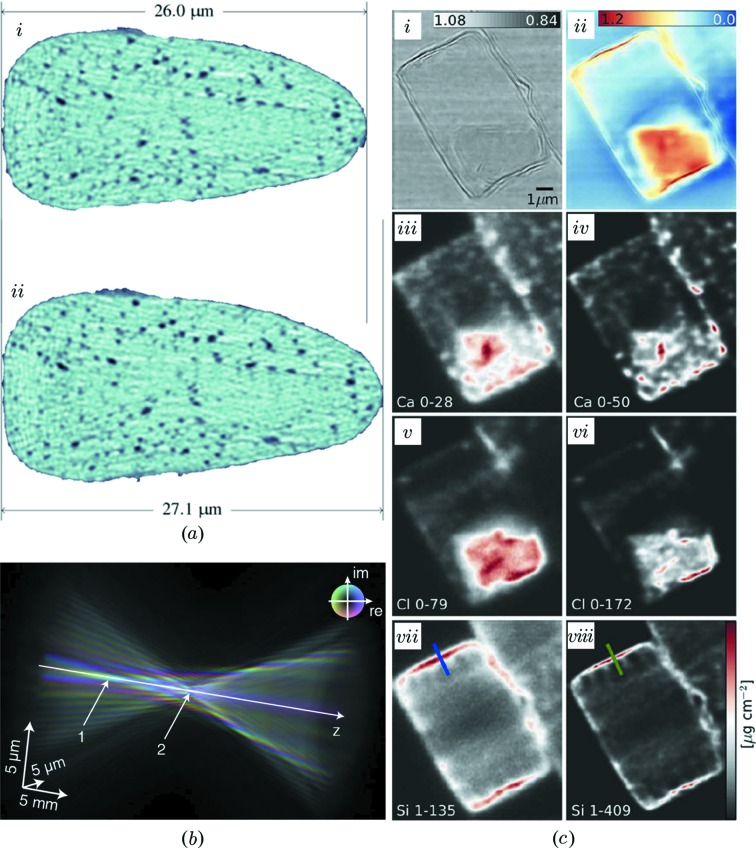
Recent results in X-ray ptychography. (*a*) *In situ* ptychography on a silk fiber whose response to a change in humidity was investigated. [Reproduced from Esmaeili *et al.* (2013 ▶).] (*b*) First application of ptychography at a free-electron laser. Complex wavefield of a nanofocused X-ray free-electron laser beam. Colors indicate the local phase; amplitude is encoded by brightness. [Reprinted by permission from Macmillan Publishers Ltd: Scientific Reports (Schropp *et al.*, 2013 ▶), copyright 2013.] (*c*) The co-reconstruction of the illumination function allows ptychography to corroborate, for instance, fluorescence microscopy by yielding the input to a deconvolution of the point spread. (i) Amplitude and (ii) phase of the object’s transmission. Imaged here is a freshwater diatom *Cyclotella meneghiniana*. (iii)–(iv) Ca fluorescence with the resolution corresponding to the size of the illumination (iii) or after iterative deconvolution (iv). Similarly, (v)–(vi) showing the Cl distribution and (vii)–(viii) the Si distribution. [Reproduced from Vine *et al.* (2012 ▶).]

By now, ptychographic X-ray tomography has moved beyond the mere demonstration status with recent applications including cement paste composition (Trtik *et al.*, 2013 ▶), marine coating percolation properties (Chen *et al.*, 2013 ▶), silk fiber hydration (Esmaeili *et al.*, 2013 ▶), Fig. 4(*a*) ▶, and carbon fibers characterization (Diaz *et al.*, 2014 ▶).

Another highly promising direction is the combination of spectroscopy with ptychographic imaging, providing elemental maps or even chemical sensitivity (Beckers *et al.*, 2011 ▶; Takahashi *et al.*, 2011 ▶; Maiden *et al.*, 2013 ▶; Hoppe *et al.*, 2013 ▶), Fig. 2(*c*) ▶. In fact, in many instances the access to both the absorption and the refractive phase decrement, β and δ, respectively, improves reconstruction quality and allows inferences on the composition even without the need to scan the incident energy (Clark *et al.*, 2010 ▶; Yan *et al.*, 2013 ▶; Jones *et al.*, 2013*a* ▶).

Beyond the interest in high-resolution and high-sensitivity sample imaging, the ability to reconstruct the probe function appeared early on as much more than a convenient by-product to improve reconstruction quality. It quickly became a reason in itself to carry out ptychographic experiments as an excellent mean of characterizing the focusing properties of various optics (Guizar-Sicairos & Fienup, 2009 ▶; Kewish *et al.*, 2010 ▶; Schropp *et al.*, 2010 ▶; Vila-Comamala *et al.*, 2011 ▶; Hönig *et al.*, 2011 ▶; Huang *et al.*, 2013 ▶). Such detailed characterization of the illumination of the sample allows further improvement also of non-ptychographic scanning-probe measurements, such as fluorescence mapping, as demonstrated by Vine *et al.* (2012 ▶), Fig. 4(*c*) ▶, and has recently been used for mapping the focus of compound refractive lenses at a free-electron X-ray laser (Schropp *et al.*, 2013 ▶), Fig. 4(*b*) ▶.

Other techniques stemming from ptychography, while still in their infancy, offer great promises and are being actively developed. This is the case for near-field ptychography (Stockmar *et al.*, 2013 ▶), which puts to work ptychography’s algorithmic tools to solve the phase retrieval problem of holographic measurements. Another approach emulating ptychography’s transition from ‘single-shot CDI’ is Bragg ptychography, which uses far-field measurements of the diffraction from a Bragg reflection of a, typically micrometer-sized, crystal as it is scanned through a coherent illumination. As in Bragg CDI (Williams *et al.*, 2003 ▶; Robinson & Harder, 2009 ▶), full rocking curves are collected, resulting in three-dimensional diffraction data that depend both on the crystallite shape transform and the illumination profile (Godard *et al.*, 2011 ▶; Hruszkewycz *et al.*, 2012 ▶, 2013 ▶; Huang *et al.*, 2012 ▶; Takahashi *et al.*, 2013 ▶).

### 2.2. Methodological developments   

Recent theoretical and algorithmic progress has also played an important role as a supplement and support to the experimental achievements. In particular, a current trend has been to relax dependence on *a priori* knowledge, frequently corresponding to what was initially assumed to be essential conditions for the success of an experiment.

Soon after the demonstration in 2004 that image reconstruction was feasible even with a relatively sparse dataset (Faulkner & Rodenburg, 2004 ▶) came the realisation that such sparse datasets were also sufficient for the simultaneous retrieval of the illumination function (Guizar-Sicairos & Fienup, 2008 ▶; Thibault *et al.*, 2008 ▶; Maiden & Rodenburg, 2009 ▶). More recently, it was shown that even relatively strong partial-coherence effects could be accounted for, either through a blind deconvolution approach (Clark & Peele, 2011 ▶) echoing earlier work using Wigner distribution formalism (Rodenburg & Bates, 1992 ▶; Chapman, 1996 ▶, 1997 ▶) or using a modal decomposition (Thibault & Menzel, 2013 ▶), also following previous investigations in similar context (Whitehead *et al.*, 2009 ▶). The culmination of this progress, termed ‘information multiplexing’, is the demonstration that even spectral diversity in the incident beam and in the sample response can be recovered in ptychographic datasets (Batey *et al.*, 2013 ▶).

In a somewhat similar fashion, but using fundamentally different approaches, it has been shown that propagation effects within thick samples could also be accommodated following a multi-slice reconstruction approach (Maiden *et al.*, 2012*a* ▶; Suzuki *et al.*, 2014 ▶), allowing three-dimensional information to be extracted from two-dimensional data without the need for tomographic methods. This does not only extend the depth of field, but allows accounting for multiple-scattering effects as well.

Other promising types of relaxation have also been introduced, from the possibility to achieve super-resolution (Maiden *et al.*, 2011 ▶), provided a strongly scattering illumination, to the practicability of violating Nyquist sampling conditions at the detector plane (Zhang *et al.*, 2007 ▶; Edo *et al.*, 2013 ▶).

Of a different flavor, but arguably having the largest impact, is the mounting evidence that positions of the illumination relative to the sample can also be recovered, or at least refined, from a ptychographic dataset (Guizar-Sicairos & Fienup, 2008 ▶; Beckers *et al.*, 2012 ▶; Zhang *et al.*, 2013 ▶; Maiden *et al.*, 2012*b* ▶; Tripathi *et al.*, 2014 ▶). This result has far-reaching consequences since mechanical reproducibility and drift remain central concerns for the endeavor of attaining ever finer reconstruction resolution.

## 3. Ptychography with diffraction-limited sources   

The synchrotron community is now preparing for the advent of fourth-generation X-ray sources (Hettel, 2014 ▶). Many third-generation synchrotrons are undergoing or planning upgrades to be converted into high-brightness storage rings. Plans to build energy-recovery linacs are also being considered seriously. And although they have very different characteristics, X-ray free-electron lasers are already having a profound impact on the way X-ray science is being conducted.

The key common attribute to all such new sources is their significantly lower emittance, which is a measure of the phase space volume occupied by the source. Whereas lowering the emittance does not affect the total flux of a source, it directly affects the beam brilliance and its coherent properties. Specifically, the coherent portion of the total flux *F* is given by 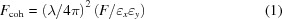
where λ is the X-ray wavelength and 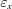, 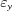 are the emittances in the horizontal and vertical directions. The beam is seen to become essentially completely coherent at the diffraction limit, *i.e.* when the emittance reaches its lowest bound, 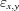 = 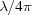. The vertical emittance of most third-generation storage rings already reaches the diffraction limit in the soft X-ray regime. Therefore, the projected improvements that will have the highest impact are those aiming at reducing the horizontal emittance.

To put into context the potential improvements brought about by fourth-generation sources, let us first consider the conditions in which the dataset presented in Fig. 3 ▶ was obtained. The total flux at the sample position of the cSAXS beamline is about 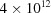
 photons s^−1^ (0.01% bandwidth)^−1^ at 6.2 keV. With an emittance of about 30 nm rad and 400 pm rad in the horizontal and vertical directions, respectively, one finds that the coherent flux available for a ptychography experiment is about 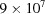
 photons s^−1^ (0.01% bandwidth)^−1^. This value is consistent with a mixed-state characterization of the beam (Thibault & Menzel, 2013 ▶), which gave about 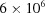
 photons s^−1^ for a beam that was sliced down vertically to a 80 µm aperture.

The promise of diffraction-limited sources is ultimately to offer an increase in coherent flux of two to three orders of magnitude, assuming the reduction in emittance is accompanied by other advances, such as higher ring current, improved insertion devices, and more efficient optics. Such technological leap needs to further be accompanied by appropriate detector and positioning technology. To illustrate the potential benefits of a thousand-fold increase in coherent flux, we extrapolate the state-of-the-art figures. We thus expect improvements along two axes that we simply label *quantity* and *quality*.

### 3.1. Gains in quantity   

The *quantity* axis describes the trivial increase in throughput for ptychography, either through an expansion of the reconstructed sample volume or through shorter scan times. In the first case, a thousand-fold increase in photons usable for an experiment would allow us to move from the current ∼Gigavoxel acquisitions towards Teravoxel tomograms in the same acquisition time. Thus, statistically relevant sample volumes of hierarchical structures, such as neural networks, cement pastes or porous catalyst supports, can be imaged with an easier compromise between resolution and field of view.

Characterizing such a larger volume in the acquisition time currently needed for fewer voxels simply entails conducting ptychography faster, and the same argument reduces the acquisition time for the measurement represented in Fig. 3 ▶ to a mere 30 s. Even when adopting an optimistic viewpoint on the technical developments of the next decade, such a short time for a complete ptychography dataset of many million resolution elements represents a significant challenge. Motion of the sample along three different axes will probably have to be simultaneous and uninterrupted, thus producing a unique three-dimensional scan. With hundreds or thousands of Gigavoxel tomograms collected within a single day, statistically significant ensembles can be surveyed, for instance to study phenotypic variations and drug-dependent effects at the sub-micrometer scale (Mader *et al.*, 2013 ▶).

The most limiting factor to reach such throughput, however, is probably the development of X-ray detectors that can withstand and efficiently measure photon counts for such high flux densities and at kHz readout rates (Schmitt & Denes, 2014 ▶). Photon-counting detectors, which have been instrumental in the success of X-ray ptychography, are fundamentally limited to count rates 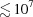
 photons per second and per pixel, a rate that is already reached nowadays under certain experimental conditions. To circumvent this limitation, either a larger number of pixels will be needed or it will be necessary to revert to integrating or ‘smart’ detectors that operate in a hybrid counting/integrating mode. Such detectors are currently being developed, thanks in part to the specialized needs of X-ray free-electron lasers. Other ways to optimize the use of detector technology will involve methodological expansions, for instance by pushing further the idea of using focusing optics or diffusers (Vine *et al.*, 2009 ▶; Guizar-Sicairos *et al.*, 2012 ▶; Maiden *et al.*, 2013 ▶) to spread the signal to less used parts of the detector. Working with Fresnel (Vine *et al.*, 2009 ▶; Jones *et al.*, 2013*b* ▶) or near-field (Stockmar *et al.*, 2013 ▶) diffraction also typically decrease the demands in detector dynamic range.

Obviously, higher data acquisition rates have consequences on the information technology side. Next to high-performance optimization of reconstruction codes, many facilities are also upgrading their infrastructure to support fast and high-capacity storage, as well as the computing resources needed to complete the data analysis and reconstructions on site and, ideally, in real time.

### 3.2. Gains in quality   

The second axis, which we labeled *quality*, pertains to the use of higher flux to push resolution and sensitivity. Three orders of magnitude more coherent flux translates into the same fluence per voxel for voxels ten times smaller along all dimensions. This simplistic view on resolution increase, however, neglects the fact that the feature size distribution of most natural samples follows a sub-cubic power law. In terms of far-field scattering this translates into a radial decay of the scattering power in 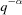, with 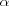 ≃ 4. Taking only this consideration into account, a full order of magnitude increase in resolution requires a 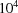-fold flux increase, which the novel sources shall render feasible.

Pushing resolution down to a few nanometers is extremely challenging and, perhaps, coherent flux might prove one of the easier challenges to solve. For instance, to conserve coherence and avoid blurring and distortions, vibrations and drifts of many components of the instrument must be reduced to below a nanometer. While challenging, until now there is no indication that this type of improvement was approaching any fundamental limit. More importantly, the very physics of interaction between the X-ray beam and the sample limit the resolution that can possibly be achieved unless diffract-before-destroy schemes (Neutze *et al.*, 2000 ▶; Seibert *et al.*, 2011 ▶) can be brought to bear, which in turn pose their specific problems for imaging. Thus, we consider the onset of radiation damage setting a strong sample-dependent bound on the achievable resolution. For organic samples, it has been argued that such a bound is roughly 10 nm (Howells *et al.*, 2009 ▶).

Since coherent flux scales sensitively with photon energy, equation (1), most coherence-depending imaging at synchrotrons has been limited to photon energies <10 keV thus far. A reduction in emittance widens the applicability of CDI techniques in the direction of hard X-rays, and with sufficiently brilliant sources coherent sub-Ångstrom wavelength X-rays could be used to achieve high-resolution tomography; specifically, local tomography on bulky samples could become routinely feasible thanks to hard X-rays’ increased penetration power.

To summarize, we expect that the novel X-ray sources will have a profound impact on both the way ptychography is conducted and the systems it can be applied to. New frontiers will be reached along both the quality and quantity axes. However, it should be kept in mind that these improvements can materialize only as long as other technical improvements follow. In particular, a sustained effort in X-ray detector development is an essential condition to reap fully the benefits offered by diffraction-limited storage rings.

## 4. Conclusion and outlook   

In this article, we have focused our attention primarily on the most immediate, and admittedly, to the specialist, most obvious, future steps ptychography will be taking as the average brilliance of synchrotron sources is pushed to new climaxes. But given these improving experimental conditions, is new science around the corner? Beyond its important though incremental improvements, is ptychography about to experience a *qualitative* jump, opening radically new fields of investigation?

On the theoretical side, ptychography’s active development and sustained rate of successes hints at its potential as an important player in contemporary questions on data acquisition strategies, information content and feature extractions. In the last few years, ptychography has been constantly redefining how ‘messy’ an experiment can be while delivering acceptable results, echoing conclusions reached in other context (Candès & Wakin, 2008 ▶). The field is also quickly reaching a point where ‘big data’ approaches need to be considered, not unlike present high-speed tomography, particle physics and XFEL experiments.

In the case of applications, the next important step could be ‘four-dimensional imaging’, where the fourth dimension would be the X-ray energy, an absolute time, or a time delay as in pump–probe experiments. More exotic quantities such as the decoherence caused by the sample could also be mapped in three or four dimensions. The interplay between the experimental and theoretical sides of ptychography will remain a key ingredient for the success of these future endeavors.
