# Coherent imaging at the diffraction limit. Erratum

**DOI:** 10.1107/S1600577515001575

**Published:** 2015-02-27

**Authors:** Pierre Thibault, Manuel Guizar-Sicairos, Andreas Menzel

**Affiliations:** a Department of Physics and Astronomy, University College London, UK; b Paul Scherrer Institut, 5232 Villigen PSI, Switzerland

## Abstract

A correction to the article by Thibault *et al.* [*J. Synchrotron Rad.* (2014), **21**, 1011–1018] is given.

Fig. 4(*c*) in the paper by Thibault *et al.* (2014 ▶) was reproduced with permission from Vine *et al.* (2012 ▶); however, the incorrect article reference was used. The correct reference is shown below.
